# Assessment of the spatial accessibility to health professionals at French census block level

**DOI:** 10.1186/s12939-016-0411-z

**Published:** 2016-08-02

**Authors:** Fei Gao, Wahida Kihal, Nolwenn Le Meur, Marc Souris, Séverine Deguen

**Affiliations:** 1 EHESP Rennes, Sorbonne Paris Cité, Rennes, France; 2 Inserm, UMR IRSET Institut de recherche sur la santé l’environnement et le travail, Rennes, 1085 France; 3 EHESP, EA 7348 MOS Management des organisations en santé, Rennes, France; 4 IRD, UMR_D 190 “Emergence des Pathologies Virales” (IRD French Institute of Research for Development, Aix-Marseille University, EHESP French School of Public Health), Marseille, France; 5 Department of Quantitative Methods for Public Health, EHESP School of Public Health, Avenue du Professeur Léon Bernard, 35043 Rennes, France

**Keywords:** Potential spatial accessibility of healthcare professionals, E2SFCA, Geographic information systems, Spatial analyses, Pregnant women, Department of Nord

## Abstract

**Background:**

The evaluation of geographical healthcare accessibility in residential areas provides crucial information to public policy. Traditional methods - such as Physician Population Ratios (PPR) or shortest travel time - offer only a one-dimensional view of accessibility. This paper developed an improved indicator: the Index of Spatial Accessibility (*ISA*) to measure geographical healthcare accessibility at the smallest available infra-urban level, that is, the Îlot Regroupé pour des Indicateurs Statistiques.

**Methods:**

This study was carried out in the department of Nord, France. Healthcare professionals are geolocalized using postal addresses available on the French state health insurance website. *ISA* is derived from an Enhanced Two-Step Floating Catchment Area (E2FCA). We have constructed a catchment for each healthcare provider, by taking into account residential building centroids, car travel time as calculated by Google Maps and the edge effect. Principal Component Analyses (PCA) were used to build a composite *ISA* to describe the global accessibility of different kinds of health professionals.

**Results:**

We applied our method to studying geographical healthcare accessibility for pregnant women, by selecting three types of healthcare provider: general practitioners, gynecologists and midwives. A total of 3587 healthcare providers are potentially able to provide care for inhabitants of the department of Nord. On average there are 92 general practitioners, 22 midwives and 21 gynecologists per 100,000 residents. The composite *ISA* for the three types of healthcare provider is 39 per 100,000 residents. A comparative analysis between *ISA* and physician-population ratios indicates that *ISA* represents a more even distribution whereas the physician-population ratios show an ‘all-or-nothing’ approach.

**Conclusion:**

*ISA* is a multidimensional and improved measure, which combines the volume of services relative to population size with the proximity of services relative to the population’s location, available at the smallest feasible geographical scale. It could guide policy makers towards highlighting critical areas in need of more healthcare providers, and these areas should be earmarked for further knowledge-based policy making.

## Background

Today, all countries have implemented public health strategies in order to prevent disease and contribute to reducing health inequalities. Health inequalities originate through several factors - including the organization and management of space which could vary between socioeconomic groups. Indeed, the under-resourcing of healthcare facilities, the lack of continuity of care, and fragmentation of care across providers, for example, could be barriers to effective healthcare provision [1] and could contribute to increasing health inequalities. Access to health care, as one potential driver of health inequalities, is at the heart of public health policy and is internationally recognized as a key goal in meeting the essential health needs of individuals [2–5]. However, equitable access has proved difficult to achieve [6].

Healthcare access is a multidimensional concept that includes: availability of care, the ability to get to and pay for available care, or to seek and utilize available care. It involves financial accessibility, availability, acceptability, and geographical accessibility [7]. The accessibility of health services can then be classified into two main categories: potential accessibility (ease of accessing services based on existing conditions) and revealed accessibility (actual use of health care services in a given location). These two types of accessibility have a particular meaning in the French context. Indeed, the French health care system is generally recognized as offering one of the world’s best public health care services. Basic treatment is free to all French residents, thanks to mandatory public health insurance (Social Security); the government has taken responsibility for the financial and operational management of health insurance. The fact that financial constraints do not exist to the same extent in the other countries, makes it all the more important to assess potential access to health care - and this could be the basis upon which to estimate revealed access. This paper will focus on measuring potential spatial accessibility to health care professionals.

The impact of geographical location on health is increasingly under examination. Various studies undertaken in France and other countries have shown an unequal distribution of health service resources [8]. Evaluating geographical healthcare accessibility in residential areas provides crucial information to public policy in terms of planning service provision. For example, it allows the identification of areas having lower or higher levels of access. Traditional methods used to measure spatial healthcare accessibility are physician-population ratios, or distance/travel time to the nearest healthcare service. These may be easily calculated and interpreted, but they are limited and often provide only a one-dimensional view of accessibility [9]. For instance, the physician-population ratio resulting from the ratio of health capacity to population within an area (generally referring to an administrative area) gives a misleading picture of spatial accessibility [10]. The fundamental weaknesses of this indicator are well recognized [11–13]; it ignores potential interactions across borders as well as the unequal spatial distribution of health care professionals within a given spatial unit. The ‘shortest path’ approach ignores supply availability, because where there is more than one service to choose from, people are able to bypass the nearest service [9].

Recent developments in the field of healthcare service spatial accessibility have emerged in international research, and have converged towards the Enhanced Two-Step Floating Catchment Area (E2SFCA) method [14], which provides a summary measure of two important and related components of access: firstly, the volume of services provided relative to the population’s size and secondly the proximity of services provided relative to the population’s location. Applied by *Institut de recherche et de documentation en économie de la santé (French Research Institute in Health Economic)* [15] in 2011, an indicator named the localized potential accessibility (Accessibilité potentielle localisée) has been constructed measuring at the municipality level [16]. However, in their work, the authors considered that both health professionals and inhabitants lived at the center of the municipality ignoring the spatial variability of their locations; point particularly important in studies conducted at a small spatial unit.

In this context, our research aims to investigate the territorial healthcare access inequalities by developing an indicator named *ISA.* This index provides a measure of the spatial accessibility to healthcare service/professionals, considered separately (just general practitioners, for instance) or combined in order to integrate several healthcare professionals involved in the course of a patient’s healthcare pathway.

In order to illustrate the different steps followed in constructing the *ISA*, the population of pregnant women was selected because good monitoring during pregnancy is recognized as being particularly important [17]. Indeed, pregnancy represents a crucial period during which the mother’s health and the progress of fetal development need to be monitored regularly in order to detect health events in good time [18]. A growing number of studies have demonstrated that an absence, or poor quality, of antenatal care increases the risk of prematurity and low birth weight [19, 20].

The *Haute Autorité de Santé (French National Authority for Health)* [21] has recommended that pregnant women should undergo seven antenatal examinations. The first appointment must take place before the end of the third month of pregnancy, followed by a monthly visit from the start of the second trimester. The cost of these appointments is fully reimbursed - in theory limiting poor monitoring of the women as a result of scant economic resources. This reinforces the importance of investigating spatial accessibility prior to getting involved in the details of the individual determinants of adverse birth outcomes. All these compulsory medical examinations can be carried out by a general practitioner, midwife or gynecologist – whichever is the principal contact during the pregnancy. This is why we have focused on these three types of healthcare professionals.

A rigorous methodological approach is proposed at a fine spatial scale in order to minimize aggregation errors, taking into account edge effect, both offer and demand, as well as a more precise geolocalization of professionals and patients. This indicator allows us to identify and map the spatial patterns of care access, highlighting critical areas where healthcare professionals need to be allocated, and analyse the spatial and social origins of such inequalities.

## Methods

### Study setting and statistical unit

This study was carried out in the department of Nord, located in the north of France next to the Belgian border. With a surface area of 5743 km^2^ and a population density of 456 inhabitants per km^2^, Nord covers 46 % of the Nord-Pas-De-Calais region’s land area. This department was chosen because of its geographical disparity regarding the organization of space: on the one hand, it concentrates a significant proportion of the region’s agricultural activity, with a lot of rural areas, and on the other, there are several densely populated areas close to major cities such as Lille, Roubaix, Tourcoing and Villeneuve d'Ascq.

The statistical unit we use is the French census block known as ‘IRIS’ (Ilot Regroupé pour l’Information Statistique), defined by the National Institute of Statistics and Economic Studies [22]. The population of the IRIS unit (equivalent to a residential neighborhood) is between 1800 and 5000 inhabitants. The Nord department is divided into 1346 IRIS.

### The offer: health professionals

The postal addresses of general practitioners, midwives and gynecologists were obtained from the French state health insurance website: http://www.ameli-sante.fr/ [23]. Since patients are able to overcome geographical boundaries and consult health professionals in neighboring departments, we have considered the health professionals’ offer both within and outside of the department of Nord, in order to take the edge effect into account. The edge effect occurs where a study area is defined by a border that does not actually prevent travel across the border [24] and people are free to travel beyond that border to receive healthcare goods and services. Thus, a proportion of healthcare providers of neighboring departments (such as Pas-de-Calais, Ardennes and Aisne) were also geolocalized. Service providers were represented by their geocoded professional addresses (latitude, longitude) through Batch Geocoder [http://dehaese.free.fr/Gmaps/testGeocoder.htm].

In addition, the positional accuracy of geocoded addresses is given by the variable “accuracy”. We were satisfied where “accuracy” is greater than or equal to 6, for which we have location information accurate down to street precision. Where this is not the case, we refine the address and repeat the procedure. Street level result is a precise geocode according to The Google Geocoding API [https://developers.google.com/maps/documentation/geocoding/].

### Demand: patient and residential location

Since a resident’s exact location is unknown, we needed to provide an aggregated location at IRIS level. Instead of using the IRIS centroid, the centroid of the residential buildings for each IRIS was calculated as a way of representing population groups. The map of residential buildings came from BD TOPO® and was provided by the *Institut National de l’Information Géographique et Forestière (French National Geographic Institute)* [25]. Since we took into account the edge effect, all the population living in one of the 5561 IRIS comprising the Nord and the five surrounding departments were considered for geolocalization and distance computing process. The residential building centroid calculation of was carried out in ArcGIS (Version 10.1, ESRI Inc).

### Distance computing

The travel distance between offer (healthcare providers) and demand (patient) is a key component in computing accessibility using the E2EFCA method. We chose the car travel time between the location of each health professional and the IRIS centroid (defining the location of the patients), calculated by Google Maps. Since we had more than 24 million origins (IRIS centroid)On the basis of the E2SFCA algorithm, two steps/destination (one given healthcare provider) location pairs, we used the FILENAME statement and the URL access method within SAS to access Google Maps, and extracted both the driving time and distance each time the site was accessed [26].

### Creation of the healthcare access indicator

On the basis of the E2SFCA algorithm, two steps were developed to calculate the accessibility of each IRIS to healthcare providers.

First step: for each healthcare provider at location k, we counted all patient population (Pi) locations i that were within the threshold *d*_*ik*_ from location *k*, by applying a travel time decay function *w*(*d*_*ik*_) (see next section *Decay function & travel time threshold*), which formed the catchment area of each healthcare provider at location *k*, and then computed the provider-to-population ratio *R*_*k*_ (Eq. (1))_._

Step 1: Defines provider-to-population ratio *R*_*k*_ within the catchment area

1 $$ {R}_k\kern0.5em =\kern0.5em \frac{1}{\sum_{d_{jk}\kern0.5em <\kern0.5em {d}_{max}}{P}_i\kern0.5em \ast \kern0.5em w\left({d}_{ik}\right)} $$

where:

- *P*_*i*_ is the patient population of IRIS *i* located within a distance *d*_*max,*_
- *d*_*ik*_ is the distance between IRIS *i* and healthcare provider *k* and,
- *w(d*_*ik*_*)* is the weight quantifying the travel time between IRIS *i* and healthcare provider *k.*

Second step: for each demand (patient population) location *i*, we searched all healthcare provider locations *k* that were within the threshold distance *d*_*jk*_ from location *i*, and then aggregated all the provider-to-population ratios *R*_*k*_ (derived in Step 1) at those locations *k* to get full accessibility *A*_*i*_ at each demand location *i* (Eq. (2)):

Step 2: Summing the *R*_*k*_ scores (defined in step 1) in order to calculate a location’s access *ISA*_*i*_

2 $$ IS{A}_i\kern0.5em =\kern0.5em {\displaystyle \sum_{{\mathrm{d}}_{\mathrm{ij}}\le {\mathrm{d}}_{max}}w\left({d}_{ij}\right){R}_k} $$

where:

- *ISA*_*i*_ represents the full accessibility of IRIS *i* to healthcare providers
- *R*_*k*_ is the provider-to-population ratio whose location *k* falls within the catchment area (*d*_*ik*_ ≤ *d*_*max*_)

The larger the *ISA*_*i*_ value, the better the accessibility to healthcare providers for a given IRIS *i*’s patient population, which can also be interpreted as more healthcare providers being available to patients within the threshold distance. The spatial analysis using the 2ESFCA method was carried out using Mysql (Version 5) and R (Version R-3.1.3) softwares.

### Decay function & travel time threshold

We defined the time threshold according to figures already published by *the French Institute for research and information in health economics* for general practitioners [16]:

- less than 5 min’ travel, we considered access to healthcare providers to be equal to 100 % (*w = 1, corresponding to full access to the healthcare providers*)
- more than 15 min’ travel, we considered access to healthcare providers to be equal to 0 (too far from residential place, *w = 0, meaning that there is no access to the given healthcare providers*)
- between 5 and 15 min, *w* is defined by a continuous decay function (3) which corresponds to partial access to healthcare providers. Previous testing suggested 1.5 was an appropriate weighting factor [27]

3 $$ w\kern0.5em =\kern0.5em \frac{\left(15-d\right)}{\left(15-5\right)}{e}^{1.5} $$

For other healthcare professionals, there was little empirical evidence to guide the choice of threshold. We based the threshold on general practitioners’ results, following the procedure set out below.

**Fig. 1:**
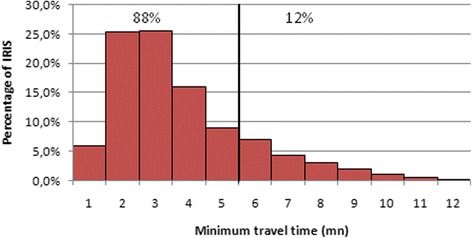
Distribution of minimum travel time for the general practitioners. The *vertical line* defines the threshold; below which the resident could reach the nearest general practitioners within 5 min, which represents 88 % of IRIS and, beyond which the time to get to the nearest general practitioners is more than 5 min. The decay function will be applied to 12 % of IRIS

- First, a function measuring the shortest travel time from each IRIS to the nearest healthcare providers was established for each type of private practitioner.
- Second, according to the “nearest travel time to general practitioner” function, we calculated the proportion of the population in Zone 1 (<= 5 min) and Zone 2 (5 to 15 min), in which the population proportion is respectively 88 % and 12 %.
- Finally, we used these proportions to define the thresholds for other general practitioners (Fig. 1).

### Composite indicator

With a view to describing the global accessibility of different kinds of health professionals involved in the course of a specific patient group’s healthcare pathways, principal component analyses were used to build a composite *ISA*. In our study, in the interest of the pregnant women, during a specific period of the pregnancy the lack of a midwife may be counterbalanced by the presence of a gynecologist. Thus, to obtain a global view of spatial accessibility to a healthcare professional during the pregnancy, we constructed a composite *ISA* that relies on principal component analysis. This approach offers the advantages of (1) taking into account the correlation between each index assessing the accessibility of the different health professionals and (2) determining the weight of each variable in the composite index.

## Results

In total, 3740 general practitioners (2590 plus 1150 located in Nord and neighboring departments, respectively), 329 obstetrical and medical gynecologists (143 and 186), and 321 midwives (218 and 103) were geolocalized at their professional postal addresses. Just eight general practitioners, and one obstetrical gynecologist, were excluded from the analysis due to incorrect, unregistered professional postal addresses.

**Fig. 2:**
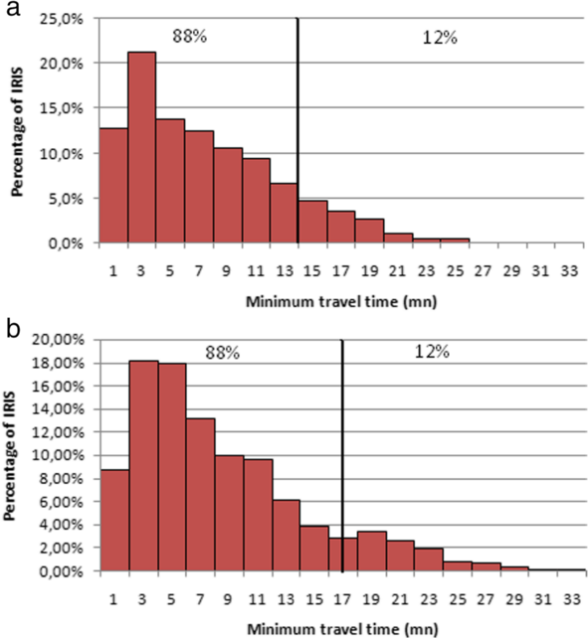
**a** Distribution of minimum travel time for gynecologists. The *vertical line* defines the threshold. **b** Distribution of minimum travel time for midwives

Figure 2a represents the IRIS distribution of minimum travel time to visit a gynecologist in the department of Nord. To obtain a proportion of IRIS in Zone 1 and Zone 2 that would match the distribution of general practitioners (88 % and 12 % of IRIS, respectively), we found the following two thresholds: 15 and 34 min. This means that within less than 15 min’ travel time, access to a gynecologist is maximal for the entire population living in these IRIS. Access to a gynecologist decreases in proportion to the decay function for the population living in the IRIS at a travel time of between 15 and 34 min. Finally, beyond 34 min, the gynecologist location is considered inaccessible for the population – in which case those gynecologists located beyond this threshold were ignored.

The IRIS distribution of minimum travel time to visit a midwife in the department of Nord is similar to that obtained for gynecologists, and the thresholds are equal to 17 and 34 min (Fig. 2b). As with the gynecologists, midwives located within travel time beyond 34 min were ignored.

In total, 3587 health professionals located in the department of Nord and neighboring departments, can potentially provide care for inhabitants of the department of Nord. The study included just 3088 general practitioners, 296 gynecologists and 203 midwives (Table 1); all other health professionals were considered inaccessible to the population living in the department of Nord. Ignoring the offer beyond the department (*edge effect*) would lead to the loss of 631 health professionals (18 % of the total number) in estimating the *ISA*. The percentage of lost health professionals reached 30 % for the midwives.

**Table 1:** Number of health professionals by medical specialty - separately for IRIS within the department of Nord and neighboring IRIS

		Department of Nord		Neighboring IRIS	
	TOTAL	Number	%	Number	%
General Practitioners	3088	2590	84 %	493	16 %
Midwives	203	143	70 %	60	30 %
Gynecologists	296	218	74 %	78	26 %
TOTAL	3587	2951	82 %	631	18 %

**Fig. 3:**
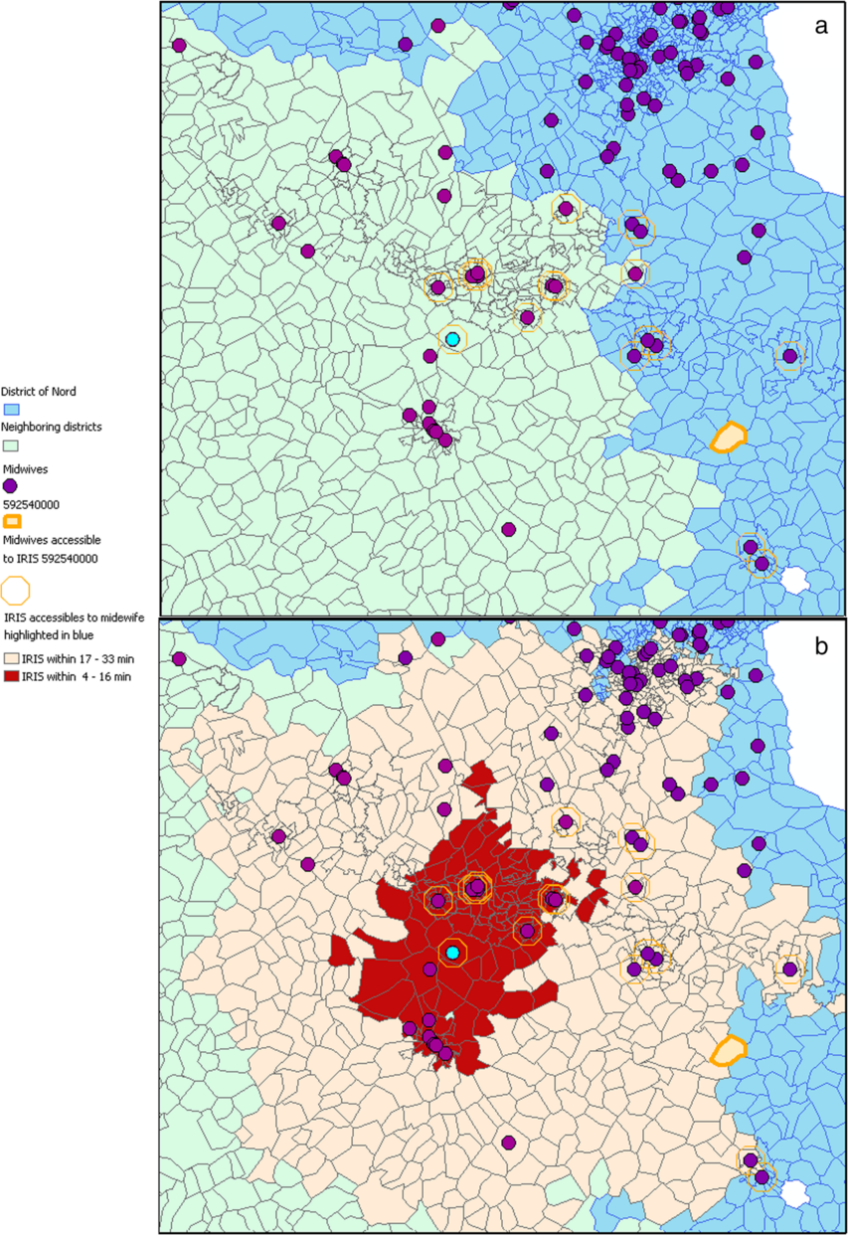
Definition of patient area- Focus on the IRIS named ‘Fressain’ (number equal to 592,540,000). IRIS of the Nord department are colored *blue*, whereas neighboring departments are *green*. The IRIS named ‘Fressain’ (number = 592,540,000) of the Nord department is highlighted in orange in (**a**) (*left*). All midwives accessible by car within 34 min of the Fressain IRIS are circled in orange. As shown in (**b**) (*right*), the patient area of the midwife highlighted in *blue* are 843 IRIS (in *red* and *pale pink* because they are accessible by car within 34 min). Accordingly, women living in the Fressain IRIS share this midwife with all the other highlighted IRIS. Depending on travel time, these 843 IRIS have different accessibility weightings (*Red Zone: 4 to 16 min, (w) = 1; Pale Pink Zone: 17 to 34 min, w = ((15-d) / (15-5))* ^*1.5*^)

The next step consists of defining the ‘patient area’ for each of the 3587 healthcare providers identified above. “Patient area” is not restricted to the 1346 IRIS of the department of Nord, since the inhabitants of Nord have to share health resources with neighboring departments. In all, 1362, 2425 and 2583 IRIS in the departments of Pas-de-Calais, Oise, Somme, Aisne and Ardennes are included in the calculation of *ISA* for general practitioners, midwives and gynecologists respectively. Figure 3 provides an illustration for the identification of healthcare providers and the definition of their ‘patient area’ for the given IRIS named ‘Fressain’ (number of IRIS equal to 592,540,000) with keys for reading.

### Spatial distribution of the accessibility index at IRIS level

**Fig. 4:**
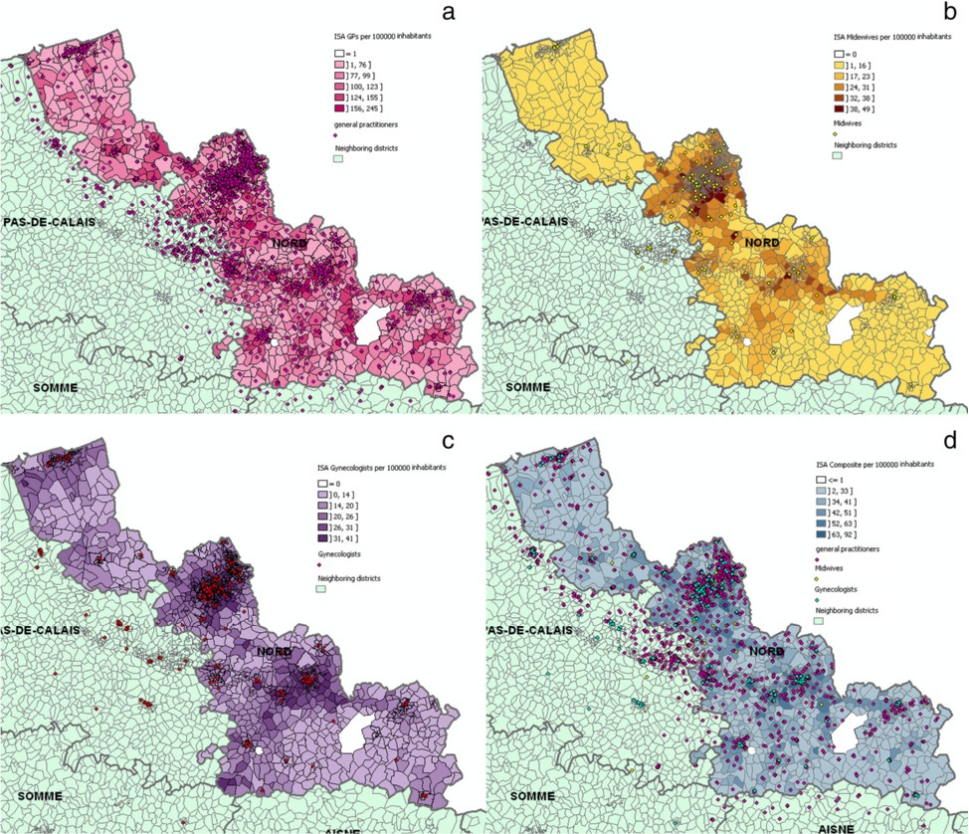
Spatial distribution at the IRIS of the Index of Spatial Accessibility general practitioners (**a**), midwives (**b**) gynecologists (**c**) considered separately, and combined in (**d**) the composite index

Spatial distributions of the accessibility index for general practitioners (a), midwives (b) gynecologists (c) considered separately, and combined in the composite index (d) are presented at IRIS level in Fig. 4. For each map, neighboring departments are colored in green while the department of Nord is colored with graduated approach, which represents different scales of *ISA* and is expressed in 100,000 inhabitants. Except the first class corresponding to the minimum value, *ISA*’s classification in 5 groups is based on the Jenks’ Natural Breaks algorithm, which assigns values to a given number of classes with the objective of minimizing variances within classes while maximizing between class means.

**Fig. 5:**
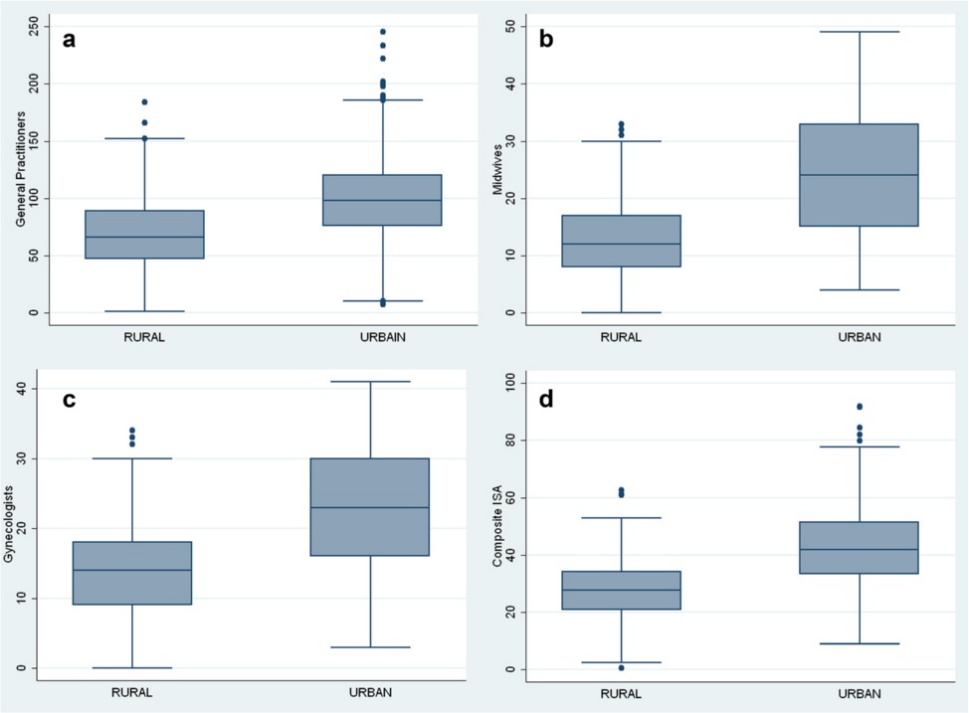
Distribution at the IRIS of the Index of Spatial Accessibility for general practitioners (**a**), midwives (**b**) gynecologists (**c**) and composite index (**d**) on an urban and rural basis

The maps reveal a non-equitable distribution of health professionals within the department of Nord at IRIS level, visible thanks to the ‘patchwork’ of color. The fact that general practitioners hugely outnumber midwives and gynecologists has repercussions for the value of spatial autocorrelation, so that (I-moran = 0.5617, *p*-value = 0.00) is lower than for either gynecologists (I-moran = 0.8805, p-value = 0.00) or midwives (I-moran = 0.9329, *p*-value = 0.00). Indeed, Fig. 4b and c reveal a similar pattern, with the highest accessibility level being in the department’s most urban area: Lille Metropolitan Area and major cities such as Roubaix, Valenciennes and Villeneuve d'Ascq. The box plot (Fig. 5) shows that the *ISA* in urban area is higher than that in rural area for three types of healthcare professionals, and there is also much more variation of the IRIS among urban areas. It is interesting to note that among 474 IRIS whose *ISA* composite belongs to the lowest natural breaks class (<33), 53 % of them belong to urban-type.

### Comparison of the *Index of Spatial Accessibility* with the physician-population ratio

The results obtained using the *ISA* and the PPR are very similar (Table 2): on average, there are 92 and 89 general practitioners per 100,000 inhabitants; 21.6 and 21 midwives per 100,000 women inhabitants aged between 15 and 44; and 20.7 and 21.5 gynecologists per 100,000 women inhabitants living in the department of Nord for both indicators, respectively. The two composite indexes are also very similar. However, differences emerge when comparing the extrema (minimum and maximum) and variability (standard deviation) of the two distributions: the PPR indicator shows ‘all-or-nothing’ access to health professionals. As an illustration, the maximum number of general practitioners is multiplied by ten when the offer is quantified by the PPR in comparison with the *ISA*.

**Table 2:** Descriptive statistics of the *Index of Spatial Accessibility* and of the Physician Population Ratio separately, by medical specialty and combined in a composite index – Department of Nord (expressed for 100,000 inhabitants)

	Index of spatial accessibility			Physician population ratio		
	Min	Mean (Sd^a^)	Max	Min	Mean (Sd^a^)	Max
General Practitioners	1	92.5 (35.1)	245	0	89. 3 (127.6)	2032
Midwives	0	21.6 (11.1)	49	0	21.0 (101.2)	1344
Gynecologists	0	20.7 (8.6)	41	0	21.5 (112.1)	2441
Composite index^b^	0,42	39.41 (13.9)	92	0	38.1 (74.5)	1120

^**a**^Standard deviation

^**b**^Composite index resulting from the first component explaining 80 % of total variability obtained with principal component analysis

**Fig. 6:**
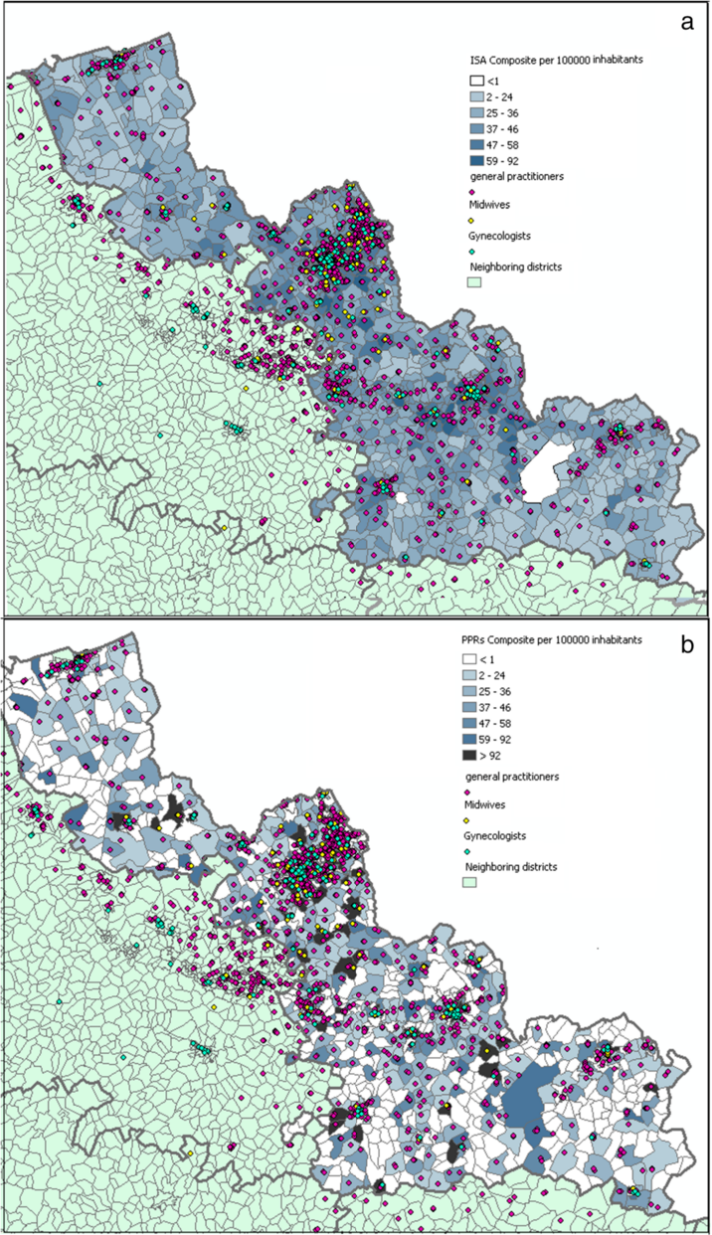
Spatial distributions of the two composite indexes at IRIS level. (**a**) *Index of Spatial Accessibility* and (**b**) the classic Physician Population Ratios

The comparison of the spatial distributions of the two composite indicators (Fig. 6) highlights the variance difference already demonstrated in Table 2; whereas the spatial distribution of the physician-population ratio reveals a large number of white or black IRIS (confirming the ‘all-or-nothing’ principle), the spatial distribution of the accessibility index is smoother, with a grey colored gradient; this is confirmed by I-Moran equal to 0.7295 being much more higher than the one obtained for the spatial distribution of the Physician Population Ratio (I-Moran equal to 0.064).

**Fig. 7:**
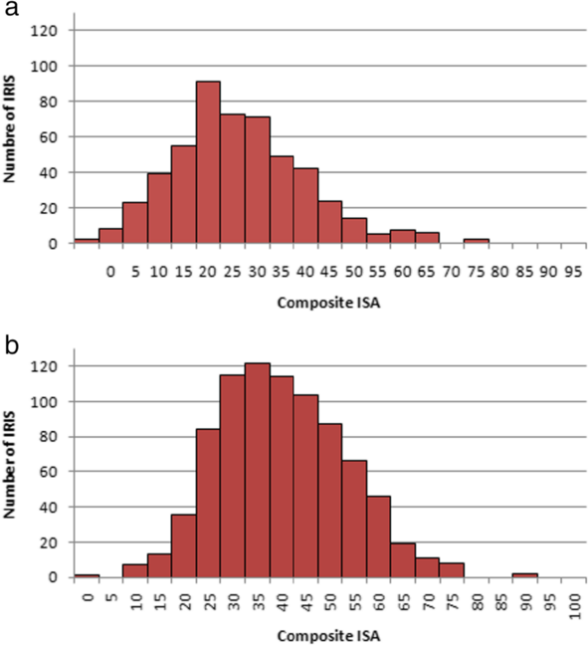
Distribution of the composite *ISA* stratified by level of the composite provider-population ratios. (**a**) composite PPRs = 0 meaning that there is no healthcare professional within the IRIS and (**b**) composite PPRs > 0 meaning that there is at least one healthcare professional within the IRIS. We can see that *ISA* is normally distributed when PPRs = 0

In Fig. 7 represents the distribution of the accessibility index for two IRIS groups: IRIS with a physician population ratio equal to 0 (meaning that there is no general practitioners, no midwife and no gynecologist located within the IRIS) and higher than 0 (at least one health professional located within the IRIS). It is particularly interesting to note the huge variability of the accessibility index distribution whereas no health professional lives in these IRIS; in other, these IRIS benefit their neighborhood.

In Table 3, we recoded the values of composite *ISA* and PPR in accordance with their quartiles for the comparison. Of the 1346 IRIS, 68 IRIS are, using the *ISA* index, classified as most advantaged (in the highest quartile, comprising between 48.31 and 91.98 healthcare professionals per 100,000 inhabitants) - whereas according to composite PPR, there are no healthcare professionals at all in these areas. Conversely, 37 IRIS having the most advantaged PPR index (comprised between 42.96 and 1119.94 healthcare professionals per 100,000 inhabitants) are considered to have poor access to health practitioners using the *ISA* index.

**Table 3:** Cross-tabulation of quartiles for the composite *ISA* and composite PPR

	*ISA* Quartiles				
Quartiles of Medical Density (PPR)	[10.41; 29.58]	[29.58; 38,16]	[38.16; 48.31]	[48.31; 91.98]	Total
0	211	130	106	68	518
[0; 16.12]	17	50	50	41	158
[16.12; 42.96]	72	93	81	89	335
[42.96; 1119.94]	37	63	100	135	335
Total	337	336	337	336	1346

## Discussion

In our study, we have drawn up a method for the construction of an indicator named *Index of Spatial Accessibility* at fine geographical scale, based on the E2SFCA algorithm, which better fits with reality by surpassing the classic limitations stressed in other studies.

Most studies examining the geographical accessibility of health care and health-related services have used various methods, including the PPR [10, 28], distance/time (Euclidean, Manhattan, or network) to the nearest healthcare professional, average distance/time to a certain number of healthcare professionals, cumulative opportunity (which counts the number of opportunities that can be reached within a travel time) [29, 30] and the gravity model [31, 32]. These methods give only a rough estimation of spatial accessibility to healthcare practitioners. As explained in the introduction, the PPR and distance/time are limited and often provide only a one-dimensional view of accessibility. Cumulative opportunity does not take into account either interaction between the population and physicians or competition between physicians [9, 33]. The gravity model is recognized as best for assessing spatial accessibility [31] but is not intuitive to interpret [26]. In the absence of detailed traffic information, the continuous distance-decay function adds complexity [28]. One important limitation of this method is that it tends to conceal health professional shortage areas - which is precisely the point that we wanted to highlight [34].

Our *ISA* indicator provides a summary measure of two important and related components of accessibility - the volume of services available relative to the population’s size, and the proximity of services available relative to the location of the population, both within the IRIS of residence and within the neighboring IRIS. We based our development on the E2SFCA method designed by Luo & Qi in 2009, which overcomes the restriction of using only pre-defined administrative regional boundaries, and differentiates distance impedance within the catchment using a decay function. Catchments are broken down into several discrete zones, with varied weightings applied to accessibility within each zone. The E2SFCA technique, like other alternative methodologies (such as PPR or the gravity model), requires demand-side population to be estimated using spatial interpolation techniques [35]. This technique is however known to lead to ecological bias, which is a particular issue when measuring neighborhood spatial accessibility to amenities. Using a fine geographical scale of analysis tends to minimize this bias and consequently improves the accuracy of estimates of spatial access to healthcare professionals [30].

Our methodology suggests several improvements in comparison to existing studies:

- Firstly, our study better describes appropriate access for a given population (here, the pregnant women) by taking into account several medical specialties (general practitioners, midwives and gynecologists) involved in the course of a patient’s health care pathway. In addition, a composite indicator is constructed to improve knowledge of the services available, and to measure overall access. As we know, an indicator that is too general is scarcely representative, because healthcare access depends on needs, and healthcare needs themselves depend on demography.
- Secondly, the French Localized Potential Accessibility indicator aggregated service providers and population at a single location, at municipality level [16]. In our study, ecological bias is reduced by precisely determining the coordinates (latitude, longitude) of each healthcare professional – rather than, as has traditionally been the case, by considering all healthcare professionals located at the centroid of the spatial unit of interest. In comparison with Localized Potential Accessibility, which has established one catchment per municipality, our method has constructed far more catchments - one per healthcare professional - which is both more precise, and flexible. We calculated the residential building centroids for each IRIS rather than the IRIS centroids, thus representing inhabitant locations more accurately, resulting in corrections of more than 4 km for several IRIS.
- Thirdly, travel time between healthcare professional and inhabitant is a critical factor in calculating accessibility using the E2FCA method. Many indicators are used to represent travel distance, such as: straight-line/Euclidian distance [36], shortest network distance [37–39], travel time, etc. [29, 30]. Travel time is a complicated indicator that is dependent on road infrastructure, transport mode and area topography [40]. Some studies have used travel time impedance, captured by combining road section lengths (obtained from Spatial Information Infrastructure) and approximate section travel speeds, which may result in a little bias for actual travel time [41]. In our study, we chose to use car travel time, as calculated by Google Maps. Compared to Euclidian distance, which is frequently used in this kind of study, car travel time is recognized as more accurate, improving the calculation. We realize that this calculation depends on car ownership. Certainly, for the most vulnerable populations, which do not have a car at their disposal, their *ISA* is overestimated, especially within urban areas –where the majority of the disadvantaged population lives. In the future, we would therefore like to integrate public transport data to obtain accurate measurement of patient travel time.
- Lastly, in the literature, the edge effect is always mentioned as a major limitation to this type of work. We were not limited by the frontier of the department of Nord, taking into account the edge effect for both professionals and patients living in neighboring departments: in other word, an inhabitant of the department of Nord could visit a doctor located in a neighboring department; and a doctor in Nord could also treat a patient coming from another department.

The results highlight a non-equitable distribution of health professionals, in particular midwives and gynecologists. The highest accessibility level tended to concentrate around the major cities. Despite a higher average level of accessibility, an important variation can be observed in urban areas, which is, 20 % of IRIS densely populated have a composite *ISA* in the lowest class.

Several limitations of our work should be addressed.

1. We considered every healthcare professional registered on the French state health insurance website (http://www.ameli-sante.fr/) [23] to work full-time at their professional postal address. If the same person is listed twice at two different addresses, without any additional information, we hypothesized that this healthcare professional works in each location half-time.
2. We used a single point as a proxy to represent the location of patient population. Despite the fact that we used the centroid of residential buildings to be accurate as possible, this may result in some ecological bias.
3. In the absence of appropriate empirical evidence, it was necessary to make a number of assumptions or estimations. This applies particularly to the definition of distance-decay function and the threshold for healthcare professionals other than general practitioners.
4. Use of a large amount of data and distance calculation prior to application of the algorithm, which is time consuming and calls for technical know-how. However, this is the price to be paid for a more accurate indicator.

## Conclusion

In conclusion, by combining availability with proximity to services, health needs and mobility, and by calculating at the smallest feasible geographical scale, our *ISA* index of access could provide a better measure of access than available methods, allowing a more equitable means of resource allocation. We used the spatial analysis and mapping capabilities of GIS to describe the spatial distribution of *ISA*, to identify the critical geographic zones of poor accessibility on an IRIS scale. This may guide policy makers towards highlighting critical areas to which additional healthcare professionals should be allocated, and these areas should be earmarked for further knowledge-based policy making. While spatial accessibility has been found to be a major and significant determinant in health outcomes [42, 43], patient access to and use of services does depend on a number of interacting factors, including socioeconomic [44] and health status, as well as perception of access.

Today, even offering universal access to health care services does not eliminate inequalities, as shown in most industrialized countries that have largely removed financial barriers to access. Different population groups such as poor people, older people, immigrants, people with disabilities, and ethnic minorities may have different health care needs and expectations [45]. One important barrier, especially for new migrants, is that they have to be French-speaking to understand precise information on universal social welfare systems (CMU) and the French network of doctors in private practice. Moreover, equity of accessibility might also be explained by a wider set of factors regarding behaviors and perceptions relating to a range of highly qualitative factors such as perceived service quality, reputation, convenience of opening hours and previous experiences. Improving access outcomes involves overcoming the social dimensions of access. Multidimensional approaches to health planning considering aspects other than spatial measures and cost have been recommended in order to identify other barriers to healthcare services. Spatial analyses provide a tool for detecting potential accessibility inequalities - but for a better understanding, further investigations and analyses of facility access and accessibility should seek to include the different dimensions relating to service access including: public perceptions, behaviors, geographical access and service quality. These were found to provide a more comprehensive analysis of health service access when considered together.

## Abbreviations

E2FCA, enhanced two-step floating catchment area; INSEE, National Institute of Statistics and Economic Studies; IRDES, Institut de Recherche et Documentation en Economie de la Santé (French Research Institute in Health Economic); IRIS, L'Îlot Regroupé pour des Indicateurs Statistiques; ISA, index of spatial accessibility; PCA, principal component analysis; PPR, physician-population ratios

## References

[1] Druss BG (2007). Improving medical care for persons with serious mental illness: challenges and solutions. J Clin Psychiat.

[2] The universal declaration of human rights. United Nations General Assembly. http://www.un.org/en/documents/udhr/index/html.

[3] Grad FP (2002). The preamble of the constitution of the World Health Organization. B World Health Organ.

[4] Department of Health and Aged Care (1999). Reforming the Australian health care system: the role of government.

[5] President’s Commission for the study of ethical problems in medicine and biomedical and behavioral research (1983). Securing access to health care: The ethical implications of differences in the availability of health services.

[6] Jatrana S, Crampton P (2009). Primary care in New Zealand: who has access?. Health Policy.

[7] Peters DH, Garg A, Bloom G, Walker DG, Brieger WR, Rahman MH (2008). Poverty and access to health care in developing countries. Ann N Y Acad Sci.

[8] Charreire H, Combier E (2009). Poor prenatal care in an urban area: a geographic analysis. Health Place.

[9] Talen E, Anselin L (1998). Assessing spatial equity: an evaluation of measures of accessibility to public playgrounds. Environ Plann A.

[10] Matsumoto M, Inoue K, Noguchi S, Toyokawa S, Kajii E (2009). Community characteristics that attract physicia ns in Japan: a cross-sectional analysis of comm unity demographic and economic factors. Hum Resour Health.

[11] Brabyn L, Skelly C. Modeling population access to New Zealand public hospitals. Int J Health Geogr. 2002; doi:10.1186/1476-072X-1-3. 10.1186/1476-072X-1-3 PMC149398 12459048

[12] Haynes R, Lovett A, Sünnenberg G (2003). Potential accessibility, travel time, and consumer choice: Geographical variations in general medical practice registrations in Eastern England. Environ Plann A.

[13] Martin D, Roderick P, Diamond I, Clements S, Stone N (1998). Geographical aspects of the uptake of renal replacement therapy in England. Int J Popul Geogr.

[14] Luo W, Qi Y (2011). An enhanced two-step floating catchment area (E2SFCA) method for measuring spatial accessibility to primary care physicians. Health Place.

[15] Institut de recherche et de documentation en économie de la santé: http://www.irdes.fr/.

[16] Barlet M, Coldefy M, Collin C, Lucas-Gabrielli V. L’Accessibilité potentielle localisée (APL) : une nouvelle mesure de l’accessibilité aux médecins généralistes libéraux. Res Inst Health Econ Lit. 2012;174.

[17] Xavier RB. and al. Reproductive risk and family income: analysis of the profile of pregnant women. J Epidemiol Community Health. 2004;58:523-527; doi:10.1136/jech.2003.011742.

[18] Faye A (2013). Social inequality and antenatal care: Impact of economic welfare on pregnancy monitoring in Senegal. Rev Epidemiol Sante Publique.

[19] Herbst MA, Mercer BM, Beazley D, Meyer N, Carr T (2003). Relationship of prenatal care and perinatal morbidity in low-birth-weight infants. Am J Obstet Gynecol.

[20] Vintzileos A, Ananth CV, Smulian JC, Scorza WE, Knuppel RA (2002). The impact of prenatal care on postneonatal deaths in the presence and absence of antenatal high-risk conditions. Am J Obstet Gynecol.

[21] Haute Autorité de santé: http://www.has-sante.fr/portail/.

[22] Institut national de la statistique et des études économiques: http://www.insee.fr/fr/.

[23] French health insurance. http://annuairesante.ameli.fr/.

[24] Fortney J, Rost K, Warren J (1999). Comparing alternative methods of measuring geographic access to health services. Heath Serv Outcomes Res Methodol.

[25] Institut national de l’information géographique et forestière: http://www.ign.fr/.

[26] Zdeb M. Driving Distances and Times Using SAS® and Google Maps. SAS Global Forum. Rensselaer, NY: University Albany School of Public Health; 2010.

[27] McGrail MR. Spatial accessibility of primary health care utilising the two step floating catchment area method: an assessment of recent improvements. Int J Popul Geogr. 2012; doi:10.1186/1476-072X-11-50. 10.1186/1476-072X-11-50 PMC3520708 23153335

[28] Ranga V, Panda P (2014). Geospat Spatial access to inpatient health care in northern rural India. Health.

[29] Talen E (2003). Neighborhoods as service providers: a methodology for evaluating pedestrian access. Environ Plann B.

[30] Apparicio P, Abdelmajid M, Riva M, Shearmur R (2008). Comparing alternative approaches to measuring the geographical accessibility of urban health services: distance types and aggregation-error issues. Int J Health Geogr.

[31] Guagliardo MF (2004). Spatial accessibility of primary care: concepts, methods and challenges. Int J Health Geogr.

[32] Martin D, Williams HCWL (1992). Market-area analysis and accessibility to primary health-care centres. Environ Plann.

[33] Fryer G, Drisko J, Krugman R, Vojir C, Prochazka A, Miyoshi T (1999). Multi-method assessment of access to primary medical care in rural Colorado. J Rural Res.

[34] Luo W, Wang F (2003). Measures of spatial accessibility to health care in a GIS environment: synthesis and a case study in Chicago region. Environ Plann B.

[35] Langford M, Higgs G. Measuring potential access to primary healthcare services: the influence of alternative spatial representations of population. The Professional Geographer. 58(3):294–306. doi:10.1111/j.1467-9272.2006.00569.x.

[36] Hewko J, Smoyer-Tomic KE, Hodgson MJ (2002). Measuring neighbourhood spatial accessibility to urban amenities: Does aggregation error matter?. Environ Plann A.

[37] Bamford EJ, Dunne L, Taylor DS, Symon BG, Hugo GJ, Wilkinson D (1999). Accessibility to general practitioners in rural South Australia. Med J Aust.

[38] Witten K, Exeter D, Field A (2003). The quality of urban environments: Mapping variation in access to community resources. Urban Stud.

[39] Apparicio P, Cloutier M-S, Shearmur R (2007). The case of Montréal’s missing food deserts: evaluation of accessibility to food supermarkets. Int J Health Geogr.

[40] Song P, Zhu Y, Mao X, Li Qi, An L. Assessing Spatial Accessibility to Maternity Units in Shenzhen, China. PLoS One. 2013; doi:10.1371/journal.pone.0070227. 10.1371/journal.pone.0070227 PMC3716609 23894622

[41] McGrail MR, Humphreys JS, Aust NZ. A new index of access to primary care services in rural areas. J Public Health. 2009; doi:10.1111/j.1753-6405.2009.00422.x. 10.1111/j.1753-6405.2009.00422.x 19811476

[42] Maheswaran R, Pearson T, Jordan H, Black D (2006). Socio-economic deprivation, travel distance, location of service, and uptake of breast cancer screening in North Derbyshire, UK. J Epidemiol Commun Health.

[43] Bin Huang MS, Dignan M, Han D, Johnson O (2009). Does distance matter? Distance to mammography facilities and stage at diagnosis of breast cancer in Kentucky. J Rural Health..

[44] Wang F, Luo W (2005). Assessing spatial and nonspatial factors for healthcare access: towards an integrated approach to defining health professional shortage areas. Health Place.

[45] Healy J, McKee M, Eds. A ccessing health care: responding to diversity. Oxford: Oxford University Press;2004.

